# Distinct Serum and Fecal Metabolite Profiles Linking With Gut Microbiome in Older Adults With Frailty

**DOI:** 10.3389/fmed.2022.827174

**Published:** 2022-04-11

**Authors:** Yan Guo, Guoqin Zhu, Fengliang Wang, Haoyu Zhang, Xin Chen, Yan Mao, Yifan Lv, Fan Xia, Yi Jin, Guoxian Ding, Jing Yu

**Affiliations:** ^1^Division of Geriatric Endocrinology, Department of Geriatrics, The First Affiliated Hospital of Nanjing Medical University, Nanjing, China; ^2^Department of Neurology, Yancheng City No. 1 People’s Hospital, Yancheng, China; ^3^Division of Geriatric Gastroenterology, Department of Geriatrics, The First Affiliated Hospital of Nanjing Medical University, Nanjing, China; ^4^Department of Breast Surgery, Nanjing Maternity and Child Health Care Hospital, The Affiliated Obstetrics and Gynaecology Hospital of Nanjing Medical University, Nanjing, China; ^5^Department of Human Biology Undergraduate, University of Toronto, Toronto, ON, Canada

**Keywords:** frailty, healthy aging, gut microbiota, metabolites, metabolomics

## Abstract

Frailty is a critical aging-related syndrome but the underlying metabolic mechanism remains poorly understood. The aim of this study was to identify novel biomarkers and reveal potential mechanisms of frailty based on the integrated analysis of metabolome and gut microbiome. In this study, twenty subjects consisted of five middle-aged adults and fifteen older adults, of which fifteen older subjects were divided into three groups: non-frail, pre-frail, and frail, with five subjects in each group. The presence of frailty, pre-frailty, or non-frailty was established according to the physical frailty phenotype (PFP). We applied non-targeted metabolomics to serum and feces samples and used 16S rDNA gene sequencing to detect the fecal microbiome. The associations between metabolites and gut microbiota were analyzed by the Spearman’s correlation analysis. Serum metabolic shifts in frailty mainly included fatty acids and derivatives, carbohydrates, and monosaccharides. Most of the metabolites belonging to these classes increased in the serum of frail older adults. Propylparaben was found to gradually decrease in non-frail, pre-frail, and frail older adults. Distinct changes in fecal metabolite profiles and gut microbiota were also found among middle-aged adults, non-frail and frail older subjects. The relative abundance of *Faecalibacteriu*, *Roseburia*, and *Fusicatenibacter* decreased while the abundance of *Parabacteroides* and *Bacteroides* increased in frailty. The above altered microbes were associated with the changed serum metabolites in frailty, which included dodecanedioic acid, D-ribose, D-(-)-mannitol, creatine and indole, and their related fecal metabolites. The changed microbiome and related metabolites may be used as the biomarkers of frailty and is worthy of further mechanistic studies.

## Introduction

Over the past century, human life expectancy has been greatly expanded, with more older people being characterized by multimorbidity and disability. Frailty has become the greatest barrier to keep older adults healthy and prolong healthy lifespan. Frailty is a geriatric syndrome marked by decreased reserve and increased vulnerability to stressors. The exponentially increasing number of frail people and frailty-associated adverse health outcomes (1) (i.e., morbidity, disability, hospitalization, institutionalization, and mortality) cause the huge burden on healthcare and social systems. Therefore, understanding the mechanisms underlying frailty and the identification of specific biomarkers for diagnosis and prognosis of frailty are imperative.

Frailty, also known as “accelerated aging” is a multi-factorial phenomenon with the pathological basis such as DNA damage, alterations in gene and non-coding RNA expression, loss of proteostasis, oxidative stress, and chromatin disruption (2). Therefore, multi-omics platforms (e.g., genomics, transcriptomics, proteomics, and metabolomics) have been developed for the analysis of complex conditions of frailty. The growing evidence has shown that all the pathological basis of aging can cause undesired metabolic reactions, and there is a “metabolic clock” that controls aging (3). Metabolomic techniques can be utilized to non-invasively identify and quantify metabolites in biological matrices (i.e., cells, tissues, and biological fluids). Furthermore, metabolomics is downstream from other omics and can dynamically assess changes in organismal function (4). A growing number of studies have shown that metabolites play a key role in physiological and pathological aging (5, 6), such as frailty (7–10). Kameda et al. (7) identified 15 markers for frailty, 6 markers for cognition, and 12 markers for hypomobility based on the metabolomic analysis. Among them, acetyl carnosine and UDP glucuronate are the common biomarkers of these three conditions. Consequently, the metabolomics has become a powerful tool to identify the biomarkers of metabolic deviations.

As reported, the metabolic capacity of the human body is mainly influenced by intestinal microbiota and the interactions with host cells (11–13). The human gut harbors a complex community of over 100 trillion microbial cells called the microbiome. Over the past decade, the increasing evidence has shown that the composition and function of the microbiome have undergone some changes with age. O’Toole and Jeffery (14) reported a shift in the microbiota toward a *Bacteroidetes*-predominated population and a loss of diversity in the core microbiota groups during aging. As an age-related condition, frailty has been proven to be associated with the gut microbiome (15–17). Ticinesi et al. (18) showed that frailty was associated with reduced microbiota biodiversity and low representation of butyrate-producing bacteria. Collectively, the intestinal microbiome and metabolites have undergone significant changes in frailty. However, less is known about the potential relationships between the gut microbiota, fecal, and blood metabolome in frailty.

In this study, we applied physical frailty phenotype (PFP) (19) to define physical frailty and performed 16S ribosomal DNA (rDNA) gene sequencing of fecal samples and liquid chromatography-mass spectrometry (LC-MS) metabolomics of serum and feces from middle-aged adults, non-frail, pre-frail, and frail older adults. We innovatively integrated multi-omics approaches for a comprehensive analysis of metabolite change related to the microbial composition during the progression of frailty.

## Materials and Methods

### Clinical Assessment

**FIGURE 1:**
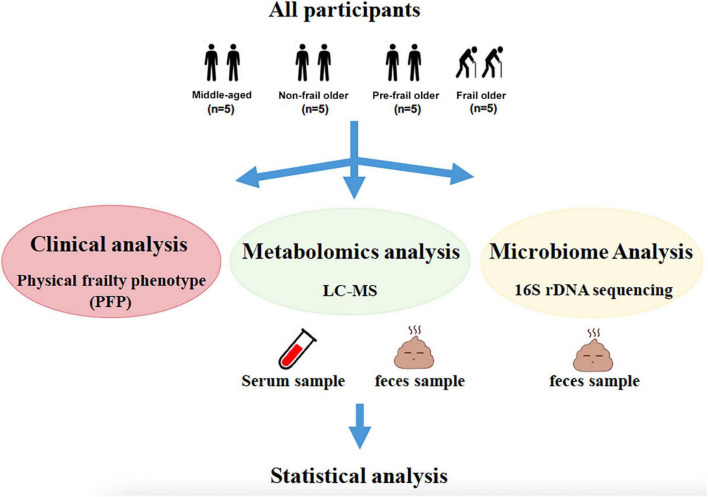
Diagram of the study protocol.

All clinical data were collected at the Department of Geriatric Endocrinology, the First Affiliated Hospital of Nanjing Medical University from 2019 to 2020. The patients were excluded who had malignant tumors, were in the acute phase of diseases, used probiotics or antibiotics within 1 month before admission and during hospitalization, or failed to complete geriatric assessment. Clinical interviews, physical examinations, and blood tests were performed for twenty participants. The demographic data that included age, sex, body mass index, healthy behavior, fall history, number of comorbidities and medications were collected. The presence of frailty, pre-frailty, or non-frailty was established according to the efficient diagnostic tool—PFP (19). The phenotypic model and diagnostic criteria of frailty are shown in Supplementary Table 1. The diagram of the study protocol can be found in Figure 1.

This study was approved by the Institutional Review Board of the Jiangsu Province Hospital and was performed in compliance with all relevant ethical regulations and guidelines. All participants provided written informed consent before recruitment.

### Feces and Blood Sample Collection

Feces samples were obtained from all recruited subjects within 3 days of admission. The participants were carefully instructed on the procedures for feces sample collection. Each sample was collected using Commode specimen collection system (Thermo Fisher Scientific, MA, United States), frozen immediately in liquid nitrogen, and then stored at –80°C before further processing.

Blood samples were collected between 08: 00 and 09: 00 a.m. by the nurses on the second day of admission after overnight fasting and collected in tubes without anticoagulant. The samples were allowed to clot for 30 min, then centrifuged at 3,000 rpm for 10 min at 4^°^C to obtain the serum, then aliquoted and stored at –80^°^C until further analyses.

### Metabolomics on Serum and Feces Samples

#### Sample Preparation

Serum samples were thawed on ice before the extraction using 100 μl serum and 300 μl solvent [methanol/ACN (1: 1)] with a 60 s vortex step before centrifugation at 4,000 g. After centrifugation, 150 μl reconstituted solution (methanol: H_2_O = 1:1, v: v) was added to 300 μl supernatant with a 60 s vortex step before centrifugation at 4,000 g for reconstitution. The supernatants were collected for metabolomic profiling by LC-MS analysis. A quality control (QC) sample was prepared by mixing and blending equal volumes (10 μl) of the supernatant of each sample to assess the analytical variability. Feces samples were thawed on ice before extraction using 25 mg feces and 800 μl solvent [methanol/acetonitrile/ACN (2:2:1)] with a 5 min grinding before centrifugation at 25,000 rpm for 15 min at 4^°^C. After centrifugation, 600 μl reconstituted solution (methanol: H_2_O = 1: 9, v: v) was added to 600 μl supernatant with a 60-s vortex step before centrifugation at 25,000 rpm for reconstitution.

#### Metabolomics Analysis

Untargeted metabolomics LC-MS analysis was carried out on a Waters 2D UPLC (Waters, MA, United States), coupled to a Q-Exactive mass spectrometer (Thermo Fisher Scientific, MA, United States) with a heated electrospray ionization (HESI) source and controlled by the Xcalibur 2.3 software program (Thermo Fisher Scientific, Waltham, MA, United States). Data were collected in both positive and negative ion modes to improve the coverage of metabolites. A Waters ACQUITY UPLC BEH C18 column (1.7 μm, 2.1 mm × 100 mm, Waters, United States) and a mobile phase consisted of 0.1% formic acid (A) and acetonitrile (B) in the positive mode and 10 mm ammonium formate (A) and acetonitrile (B) in the negative mode were used for chromatographic separation. The column was maintained at 45°C, and the gradient conditions were as follows: 0–1 min, 2% B; 1–9 min, 2–98% B; 9–12 min, 98% B; 12–12.1 min, 98% B to 2% B; and 12.1–15 min, 2% B. The flow rate was 0.35 ml/min and the injection volume was 5 μl. The mass spectrometric settings for positive and negative ionization modes were as follows: spray voltage was 3.8/-3.2 kV; sheath gas flow rate was 40 arbitrary units (arb); aux gas flow rate was 10 arb; aux gas heater temperature was 350°C; capillary temperature was 320°C. The full scan range was 70–1,050 m/z with a resolution of 70,000, and the automatic gain control (AGC) target for MS acquisitions was set to 3e6 with a maximum ion injection time of 100 ms. Top 3 precursors were selected for subsequent MS fragmentation with a maximum ion injection time of 50 ms and resolution of 17,500, and the AGC was 1e5. The stepped normalized collision energy was set to 20, 40, and 60 eV.

#### Data Analysis

Raw LC-MS data were extracted and processed following previously published protocols (20). Multivariate statistical analysis [partial least squares method-discriminant analysis, (PLS-DA)] was used to establish a relationship model between metabolites and sample groups. The univariate methods (Wilcoxon test and two-tailed Student’s *t*-test) were used to detect significantly changed metabolites and then corrected by false discovery rate (FDR) to ensure that metabolite peaks were reproducibly detected. A number of metabolites responsible for the difference in the metabolic profile scan between groups were obtained based on the variable importance in the projection (VIP) threshold of 1 from the sevenfold cross-validated PLS-DA model. By combining the univariate and multivariate statistical analyses, significantly changed metabolites between groups were acquired on the condition of *p*-value < 0.05, *q*-value < 0.05, fold-change < 0.8 or > 1.2, VIP > 1. Annotations and identifications of the metabolites were performed following the previously published protocols (20).

#### Pathway Analysis

The Kyoto Encyclopedia of Genes and Genomes (KEGG) database was used to understand the functional characteristics of differential metabolites and determine the main biochemical metabolic pathways and signaling transduction pathways involved in the metabolites. In the pathway enrichment analysis of differential metabolites, the significantly different metabolites were compared with the overall identified metabolites as a background, and the hypergeometric test was used to find significantly enriched pathway entries. Metabolic pathways with *p*-value < 0.05 were significantly enriched by differential metabolites.

### 16S rDNA Microbiome Analysis

#### 16S rDNA Sequencing

DNA was extracted from all fecal samples using MagPure Stool DNA KF kit B (Magen, Guangzhou, China) following the manufacturer’s instructions. The 16S rDNA was PCR-amplified and then sequenced on the MiSeq system (Illumina, CA, United States). The primer sequences were F: 5′-GTGCCAGCMGCCGCGGTAA-3′ and R: 5′-GGACTACHVGGGTWTCTAAT-3′.

#### Operational Taxonomic Unit Clustering

The sequence reads with a similarity greater than 97% were identified and clustered into an Operational Taxonomic Unit (OTU) using the UPARSE software (21). Then, OTU representative sequences were taxonomically classified using Ribosomal Database Project (RDP) Classifier, and the community composition was analyzed in each taxonomic rank: domain, kingdom, phylum, class, order, family, genus, and species.

#### Rarefaction Curve

A rarefaction curve was generated using the MOTHUR package (v1.31.2) (22) for richness estimations of the OTUs.

#### Diversity Analysis

Alpha diversity was performed to identify the complexity of species diversity for each sample (group). To assess the diversity in samples (groups) for species complexity, beta diversity calculations were analyzed. Alpha and beta diversities were estimated by MOTHUR (v1.31.2) and QIIME (v1.8.0) (23) at the OTU level, respectively. Sample cluster was conducted by QIIME (v1.8.0) based on the unweighted pair group method with arithmetic mean (UPGMA).

#### Functional Annotation

KEGG functions of the microbiota were predicted using the PICRUSt software (24).

#### Statistical Analysis

Statistical analysis was performed using R (v3.4.1) software. Continuous variables were presented as median (interquartile range) while categorical data were expressed as number and percentage (%). Significant differences in species and functions were evaluated with Wilcox test or Kruskal test. Linear discriminant analysis (LDA) coupled with effect size (LEfSe) was applied to evaluate the differentially abundant taxon.

### Correlation Analysis Between Metabolites and Microbiota

The Spearman’s correlation analysis based on R package (v3.4.1) was used to analyze the correlation between metabolites and microbiota. Differences were considered significant when *p* < 0.05 and | *r*| >0.5. If *r* < 0, there was a negative correlation, otherwise, there was a positive correlation.

## Results

### Characteristics of Participants

A total of twenty participants consisted of five middle-aged adults and fifteen older adults, of which fifteen older subjects were divided into three groups: non-frail, pre-frail, and frail, with five subjects in each group. The details of PFP of fifteen older adults are shown in Supplementary Table 2. Middle-aged adults were free from serious diseases and the resulting weakness, aged 40–48 years. The other three groups of older adults had similar age (non-frail subjects, median 82 years old; pre-frail subjects, median 82 years old; frail subjects, median 84 years old). Both pre-frail and frail subjects had a higher number of comorbidities than non-frail subjects, and frail subjects used a greater variety of medications than non-frail older adults. The clinical characteristics of participants are shown in Supplementary Table 3.

### Serum Metabolites Altered in Frailty

**FIGURE 2:**
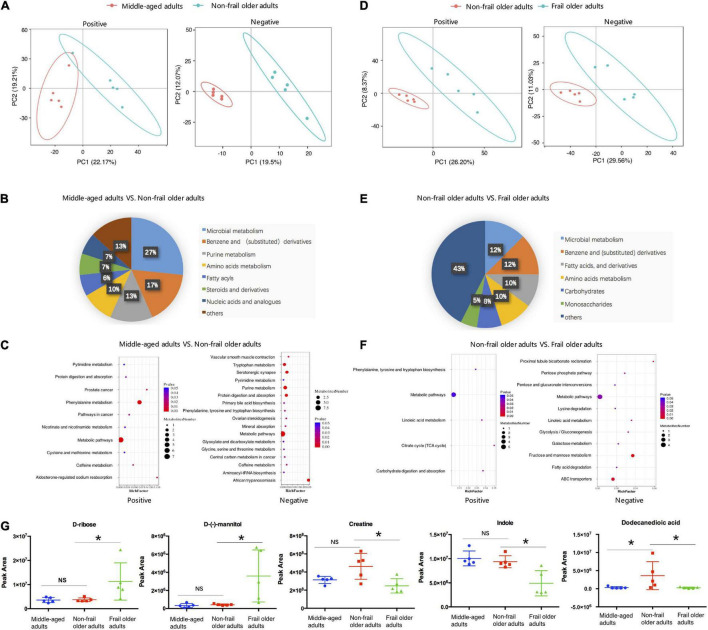
Serum metabolome analysis. **(A,D)** PLS-DA analysis of the grouped discrimination by the first two principal components (PCs) in positive (pos) and negative (neg) ion modes. **(B,E)** Pie graph of the metabolite class composition of significantly altered metabolites according to the number of metabolites in serum. **(C,F)** Bubble chart of pathway enrichment analysis of differential metabolites in positive (pos) and negative (neg) ion modes. RichFactor was the number of differential metabolites divided by all the identified metabolites annotated to the pathway. **(G)** Relative abundance of the representative differential metabolites. Peak area: relative concentrations of metabolites. *p < 0.05. Error bars represented mean ± SD.

Totally, 448 metabolites in negative ion and 1,077 metabolites in positive ion mode were identified. Raw LC-MS data are available from the MetaboLights repository (accession no. MTBLS4367). To observe the metabolic characteristics of healthy aging, we compared the serum metabolome of non-frail subjects with middle-aged adults, and PLS-DA showed the differences between the two groups (Figure 2A). The relative concentration of 41 metabolites decreased and 49 metabolites increased in the serum of non-frail subjects (*p* < 0.05, Supplementary Table 4). According to the altered metabolites with class information, healthy aging showed the changes of microbial metabolism, benzene and (substituted) derivatives, and purine metabolism (Figure 2B). Furthermore, KEGG metabolic pathway analysis based on the altered metabolites revealed the significant enrichment of 22 pathways in healthy aging, mainly including phenylalanine metabolism, purine metabolism, and tryptophan metabolism pathways (Figure 2C and Supplementary Table 5).

To analyze the metabolic characteristics of frailty, we then compared the serum metabolome of frail group with non-frail group. PLS-DA revealed a separation of the two groups based on the first two principal components (Figure 2D). Totally, 156 significantly altered metabolites were found between the two groups, with 63 metabolites upregulated and 93 metabolites downregulated in the serum of frail subjects (Supplementary Table 6). The analysis of the metabolites with class information showed that in addition to microbial metabolism, benzene, and (substituted) derivatives, the shifts in frailty mainly included fatty acids (and derivatives), carbohydrates, and monosaccharides (Figure 2E). Most of the metabolites in these classes had increased levels in the serum of frail subjects. Remarkably, propylparaben gradually decreased in non-frail, pre-frail, and frail older subjects, while other metabolites did not show a meaningful trend (Supplementary Table 7). KEGG metabolic pathway mapping showed that the altered metabolites were significantly enriched in 13 pathways, such as fructose and mannose metabolism, ABC transporters, and lysine degradation (Figure 2F and Supplementary Table 8). Relative concentrations of representative metabolites with class and pathway information, such as D-ribose, D-(-)-mannitol, creatine, indole, and dodecanedioic acid, are shown in Figure 2G. These results revealed distinct serum metabolic characteristics of healthy aging and frailty.

### Fecal Metabolites Altered in Frailty

**FIGURE 3:**
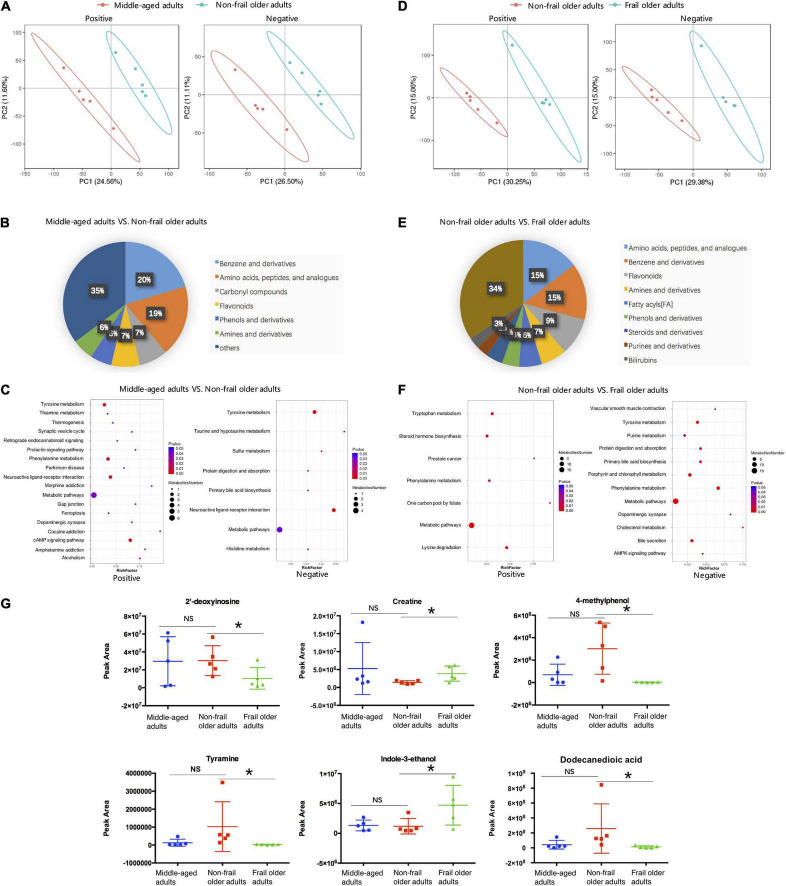
Fecal metabolome analysis. **(A,D)** PLS-DA analysis of the grouped discrimination by the first two principal components (PCs) in positive (pos) and negative (neg) ion modes. **(B,E)** Pie graph of the metabolite class composition of significantly altered metabolites according to the number of metabolites in feces. **(C,F)** Bubble chart of pathway enrichment analysis of differential metabolites in positive (pos) and negative (neg) ion modes. RichFactor was the number of differential metabolites divided by all the identified metabolites annotated to the pathway. **(G)** Relative abundance of the differential metabolites related to the serum metabolites shown in Figure 2G. Peak Area: relative concentrations of metabolites. *p < 0.05. Error bars represented mean ± SD.

Strikingly, fecal metabolomes were also clearly different in healthy aging and frailty. Totally, 1,422 metabolites in negative ion and 2,538 metabolites in positive ion mode were identified. Raw LC-MS data are available from the MetaboLights repository (accession no. MTBLS4372). PLS-DA showed a clear separation trend between middle-aged adults and non-frail subjects (Figure 3A). Compared to middle-aged group, 95 metabolites had higher and 111 metabolites had lower concentration in the feces of non-frail subjects (Supplementary Table 9). According to the class information, fecal metabolite change in healthy aging mainly included benzene (and derivatives) and amino acids, and peptides (and analogs) (Figure 3B). The differential metabolites were significantly enriched in 22 metabolic pathways using KEGG pathway analysis, including phenylalanine metabolism and tyrosine metabolism (Figure 3C and Supplementary Table 10).

As for frailty, the differences in fecal metabolites between non-frail and frail groups were showed by PLS-DA (Figure 3D). Totally, 462 metabolites significantly changed between non-frail and frail subjects, with 191 metabolites upregulated and 271 downregulated in frail elderly group (Supplementary Table 11). In addition to amino acids, peptides (and analogs) and benzene (and derivatives), the analysis of metabolites with class information displayed that the shifts in frailty mainly included fatty acyls, steroids (and derivatives), and purines (and derivatives) (Figure 3E). In the same way, KEGG pathway analysis of significantly altered metabolites revealed that 16 metabolic pathways were enriched in frailty, which included bile secretion, lysine degradation, and steroid hormone biosynthesis (Figure 3F and Supplementary Table 12). Relative concentrations of 2′-deoxyinosine, creatine, 4-methylphenol, tyramine, indole-3-ethanol, and dodecanedioic acid which had relationship with the serum metabolites in Figure 2G also changed in fecal samples of frail subjects (Figure 3G). Just like serum metabolomes, these results showed differential fecal metabolites and changes in several amino acid metabolic pathways in healthy aging and frailty.

### Taxonomic Alterations of Gut Microbiota in Frailty

**FIGURE 4:**
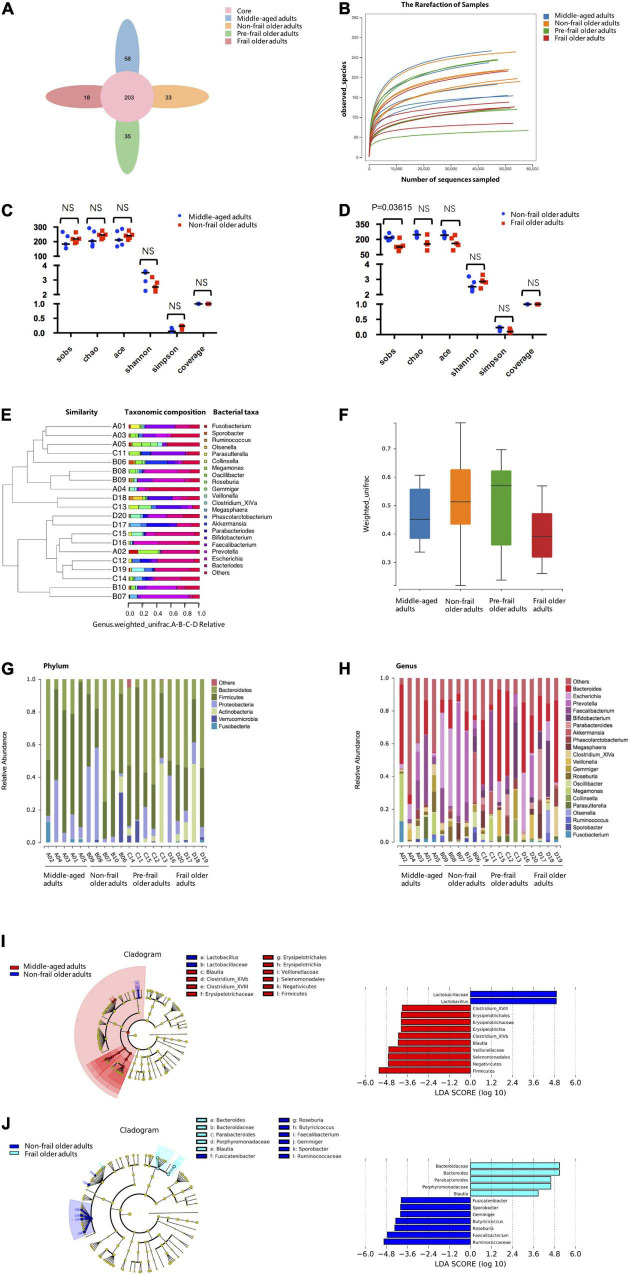
16S rDNA-amplicon sequencing analysis. **(A)** The Core-Pan diagram of OTUs distribution among four groups. **(B)** The rarefaction curve of random sequences per sample and their corresponding number of observed species. **(C,D)** Species diversity differences estimated by the observed Sobs, Chao, Ace, Shannon, Simpson, and coverage indices. **(E)** UPGMA cluster analysis of 20 samples at genus level. A01, A02, A03, A04, and A05 represented middle-aged group; B01, B02, B03, B04, and B05 represented non-frail group; C01, C02, C03, C04, and C05 represented pre-frail group; D01, D02, D03, D04, and D05 represented frail group. **(F)** Beta diversity box-plot based on weighted UniFrac analysis among groups. **(G)** The percentages of gut microbiota diversity at phylum level. **(H)** The percentages of gut microbiota diversity at genus level. **(I,J)** LDA integrated with effect size (LEfSe). Left: the phylogenetic distribution of microbiota in cladogram. Right: the differences in abundance of microbiota. Middle-aged adults vs. non-frail subjects **(I)**; non-frail subjects vs. frail subjects **(J)**. NS, not significant.

The previous study suggested that around 30% of metabolites detected in human body originated from microbiota (25). To identify the changes in gut microbiota during frailty and examine their effects on serum and fecal metabolites, we performed 16S rDNA amplicon sequencing of fecal samples from twenty participants, and the data were deposited in the BioProject database (accession no. PRJNA787524). A total of 3,574 OTUs were detected. The OTU Core-Pan diagram showed the number of common microbial taxonomies among groups and the unique taxonomies in each group (Figure 4A). There were 203 OTUs overlapped among four groups, whereas 996, 1,089, 798, and 691 OTUs belonged to middle-aged adults, non-frail, pre-frail, and frail subjects, respectively. The number of OTUs in non-frail group was much larger than that in other three groups, which indicated the most abundant microbiota of non-frail subjects. Rarefaction curve indicated the results of observed species corresponding to random sampling sequences per sample. A flat trend was observed as the number of sequences increased, which indicated that the sampling size was reasonable (Figure 4B).

To observe the differences in bacterial diversity among the groups, the sequences were aligned to estimate alpha and beta diversities. We did not find statistical differences in alpha diversity of microbiota community between middle-aged adults and non-frail subjects (Figure 4C). The Sobs index of the frail group was significantly lower than that of non-frail group [126 (6) vs. 218 (12), *p* = 0.03615] (Figure 4D). For beta diversity analysis, UPGMA cluster analysis based on the weighted UniFrac analysis was performed, and the phylogenetic distance between samples was calculated (Figure 4E). No statistical significance was found between non-frail subjects and middle-aged adults or frail and non-frail groups (Figure 4F).

Next, the composition of intestinal microbe in all groups was analyzed at phylum and genus levels, and we found considerable variabilities of gut microbiota across samples in each sample. A number of six and 21 species were the most distinct at the phylum (Figure 4G) and genus (Figure 4H) levels, respectively. At the phylum level, *Bacteroidetes* and *Firmicutes* were the two predominant phyla. The relative abundance of *Bacteroides* increased gradually in middle-aged adults, non-frail, pre-frail, and frail subjects (relative abundance was 0.202, 0.337, 0.392, and 0.448%, respectively). The relative abundance of *Firmicutes* decreased gradually in middle-aged adults, non-frail, and frail subjects (relative abundance was 0.622, 0.358, and 0.265%, respectively). At the genus level, there were 2 significant gut microbiota composition between middle-aged adults and non-frail subjects and 5 significant gut microbiota composition between non-frail and frail subjects (Supplementary Table 13). These data revealed the differences among these groups with more pronounced changes in the microbiota of frail subjects.

Furthermore, we used LEfSe to generate a cladogram to identify the specific bacteria associated with healthy aging and frailty. We identified 12 discriminatory microflora as the key discriminants for healthy aging. The potentially beneficial bacteria such as *Lactobacillaceae* (*Lactobacillus*) were significantly overrepresented [all LDA scores (log10) > 4.8] in feces of non-frail subjects, while *Firmicutes* [*Erysipelotrichia* (*Erysipelotrichales*, *Erysipelotrichaceae*, *Clostridium_XVIII*), *Clostridium_XlVb*, *Blautia*, and *Negativicutes* (*Veillonellaceae*, *Selenomonadales*)] were the most abundant microbiota in middle-aged adults (all | LDA scores (log10) | > 3.6) (Figure 4I). Similarly, 12 discriminatory microflorae were identified as the key discriminants in frailty. Frail subjects were mainly characterized by higher abundance of *Porphyromonadaceae* (*Parabacteroides*), *Bacteroidaceae* (*Bacteroides*), and *Blautia* [all LDA scores (log10) > 3.6], while non-frail subjects showed higher enrichment of *Ruminococcaceae* (*Faecalibacterium*, *Gemmiger*, *Sporobacter*, *Butyricicoccus*), *Roseburia*, and *Fusicatenibacter* [all | LDA scores (log10) | > 3.6] (Figure 4J). Taken together, these data indicated the alteration of commensal gut microbiome composition in healthy aging and frailty.

### Correlation Analysis of Metabolites and Gut Microbiota in Frailty

**FIGURE 5:**
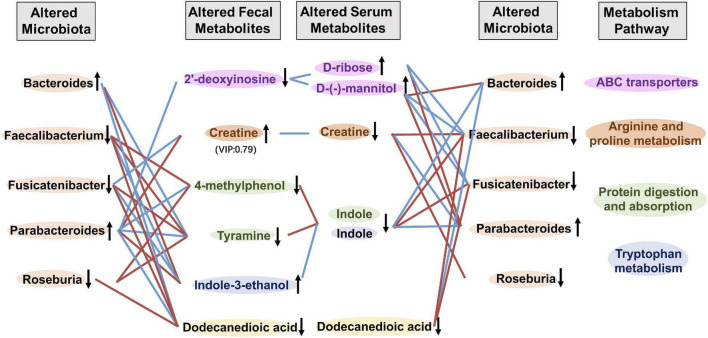
Correlation analysis between metabolites and gut microbiota. Correlation profile of altered gut microbiota, fecal and serum metabolites in frailty based on the Spearman’s correlation analysis. Fecal metabolites and serum metabolites of the same color were included in the metabolism pathway with the same color. ↑ and ↓ indicated higher and lower concentration of metabolites or abundance of microbiota, respectively. Red and blue lines indicated positive and negative correlations, respectively.

To explore the potential links between gut microbiota composition and the metabolome of frailty, we carried out inter-omics correlation analysis of the abundance of microbiota at the genus level with serum and fecal metabolites. We found that 5 serum metabolites D-ribose, D-(-)-mannitol, creatine, indole, and dodecanedioic acid had relationships with fecal metabolites and microbiota altered in frailty (Figure 5 and Supplementary Tables 14, 15). Increased serum metabolites D-ribose and D-(-)-mannitol were negatively correlated with fecal 2′-deoxyinosine and potentially beneficial bacteria such as *Faecalibacterium* and *Fusicatenibacter*. Nevertheless, as an energy source, serum creatine was negatively associated with fecal creatine and positively with *Roseburia* and *Faecalibacterium*. Decreased serum indole and the related fecal metabolites 4-methylphenol, tyramine and indole-3-ethanol were also positively associated with these potentially beneficial bacteria. Conversely, D-ribose and D-(-)-mannitol were positively related to *Bacteroides* and *Parabacteroides*, but creatine and indole were negatively related to these two microbiota that increased in frail subjects. We also found that serum and fecal levels of dodecanedioic acid, an energy substrate, were positively associated with decreased *Faecalibacterium*, *Fusicatenibacter*, and *Roseburia* and were negatively associated with *Bacteroides* and *Parabacteroides* in frailty. These results suggested that alterations of metabolite profiling and metabolic disorders in frailty might be associated with gut microbiota.

## Discussion

Serum metabolomics and gut microbiome have emerged as the exciting frontiers for understanding physiological changes associated with aging and pathophysiologic changes in frailty. In this study, we identified patterns of serum metabolites and fecal microbiota during healthy aging (middle-aged adults vs. non-frail subjects) and frailty (non-frail vs. frail subjects). Notably, partly overlapping but distinct metabolite profiles in healthy aging and frailty support the notion that frailty is an integrated spectrum of age-related disorders. According to the pie graph of the metabolites class composition and metabolite concentrations, metabolites that belong to the classes of fatty acids and derivatives, carbohydrates, and monosaccharides all increased, which might suggest protein utilization disorder or deficiency in frailty. Furthermore, we identified serum metabolites that included D-ribose, D-(-)-mannitol, creatine, and indole and their related fecal metabolites 2′-deoxyinosine, 4-methylphenol, tyramine, and indole-3-ethanol changed in frailty. Indole is involved in the metabolism of muscle-related amino acid tryptophan (26). Creatine is the major energy source in the muscle that can produce ATP rapidly to meet energy demands (27). Therefore, decreased serum levels of creatine and indole, and the changes of their related fecal metabolites also indicate the dysfunction of amino acid metabolism and protein digestion/absorption in frailty and suggest the involvement of energy deficiency from protein in the pathogenesis of frailty.

In addition, we focused on dodecanedioic acid, an even-number medium-chain dicarboxylic acids, with characteristics intermediating between glucose and fatty acids (28). It is a suitable energy substrate providing energy support during exercise, since it reduces muscle fatigue and is rapidly oxidized (29). As we have already known, muscle loss and energy deficiency resulting from aging, diseases, and weight loss are the core of the frailty cycle, which further leads to fatigue, low strength, slowness, and reduced activity (19). Moreover, Viltard et al. reported a significant increase of dodecanedioic acid in extremely long-lived naked mole rats (30), which implies its correlation with longevity. Therefore, decreased level of dodecanedioic acid may also suggest a potential mechanism related to energy deficiency in frailty.

Importantly, we observed coherent change of dodecanedioic acid in feces and serum, which suggested that decreased serum dodecanedioic acid might be due to the reduction of substrate metabolism by gut microbiota. In fact, serum dodecanedioic acid was positively correlated with *Fusicatenibacter* and *Faecalibacterium* and negatively correlated with *Bacteroides* and *Parabacteroides* in frailty. The Spearman’s correlation analysis also showed the relationships between serum metabolites D-ribose, D-(-)-mannitol, creatine, indole, and the abundance of *Faecalibacterium*, *Roseburia*, *Fusicatenibacter*, *Bacteroides*, and *Parabacteroides*. *Faecalibacterium prausnitzii* was found to be a key producer of short-chain fatty acid butyrate, which exerts an anti-inflammatory effect in the gut (31). Tongeren et al. (32) reported that *Faecalibacterium prausnitzii* significantly reduced in frail people, and the similar result was confirmed in a study conducted by Jackson et al. (17). Genera *Roseburia* and *Fusicatenibacter* were also reported to be associated with the protective effects on health (33, 34). Thus, we suppose that frailty is accompanied by a reduction in the beneficial gut microbiota that play an important role in regulating metabolites.

Since frailty status might be reversible or postponed (35, 36), it is urgent to identify the potential biomarkers of pre-frailty for the prevention and early intervention of frailty. In this study, propylparaben was found to gradually decrease in non-frail, pre-frail, and frail groups. However, due to the limitation of our sample size, propylparaben is the only coherent metabolite among non-frail, pre-frail, and frail subjects. Propylparaben is a kind of xenoestrogen and exerts neuroprotective effects in neurodegenerative diseases such as Alzheimer’s disease (37). However, an acute exposure to propylparaben may cause DNA damage in mice (38). Since its mixed directionality of association with health, the role of Propylparaben in frailty is worthy of further study.

In summary, our findings reveal distinct metabolic profiles of healthy aging and frailty. The correlation of the changes of gut microbiota and metabolites suggests the disturbances of microorganism-host metabolic balance during frailty and provides the basis for future studies on the pathogenesis of frailty.

## Data Availability Statement

The datasets presented in this study can be found in online repositories. The names of the repository/repositories and accession number(s) can be found in the article/Supplementary Material.

## Ethics Statement

The studies involving human participants were reviewed and approved by the Institutional Review Board of the Jiangsu Province Hospital. The patients/participants provided their written informed consent to participate in this study.

## Author Contributions

YG and JY wrote and revised the manuscript. YG and FW collected the samples and analyzed the data. GZ and FW analyzed the data of metabolomics. YL, FX, and YJ analyzed the data of microbiome. HZ, XC, and YM conducted the geriatric assessment. JY and GD were the guarantor of this work, as such, had full access to all the data in the study and took responsibility for the integrity of the data, the accuracy of the data analysis, and designed the experiments. All authors read and approved the manuscript.

## Conflict of Interest

The authors declare that the research was conducted in the absence of any commercial or financial relationships that could be construed as a potential conflict of interest.

## Publisher’s Note

All claims expressed in this article are solely those of the authors and do not necessarily represent those of their affiliated organizations, or those of the publisher, the editors and the reviewers. Any product that may be evaluated in this article, or claim that may be made by its manufacturer, is not guaranteed or endorsed by the publisher.
