# Supplementary material for: Distinct Serum and Fecal Metabolite Profiles Linking With Gut Microbiome in Older Adults With Frailty
Source: Front Med (Lausanne). 2022 Apr 11;9:827174. doi: 10.3389/fmed.2022.827174 (PMC9035822; doi:10.3389/fmed.2022.827174)
Supplement: Supplementary file 1 [file Data_Sheet_1.docx]

**Supplementary tables**

Supplementary Table 1. Criteria of frailty diagnosis (Physical Frailty Phenotype).

| **1. Characteristics of Frailty** | **2. Measure** |
| --- | --- |
| **Shrinking** | Deficit given to  – In the last year, have you lost more than 5 kilograms unintentionally (i.e., not due to dieting or exercise)  – BMI ≤ 18.5 kg/m² |
| **Weakness** | Average handgrip strength in dominant hand (3 trials) using JAMAR^®^ hand held dynamometer  Deficit given to  –  Males: BMI ≤ 20.6 kg/m² and strength ≤ 25.2 kg; 20.6 < BMI ≤ 25.9 kg/m² and strength ≤ 28.5 kg; BMI > 25.9 kg/m² and strength ≤ 30 kg  –  Females: BMI ≤ 20.0 kg/m² and strength ≤ 15 kg; 20.0 < BMI ≤ 24.8 kg/m² and strength ≤ 17.5 kg; BMI > 24.8 kg/m² and strength ≤ 20 kg |
| **Exhaustion** | Deficit given to  – I felt that everything I did was an effort.  – I could not get going.  A moderate amount of the time (3 - 4 days) or most of the time in the last week |
| **Slowness** | Walking speed (4 m test, usual pace, two trials, taking a short time)  Deficit given to  – Males: Walking time ≥ 5 s  – Females: Walking time ≥ 6.7 s  – The pace test could not be completed due to physical reasons |
| **Low activity** | Minnesota Leisure Time Activity Questionnaire (past 2 weeks): walking, chores (moderately strenuous), mowing the lawn, raking, gardening, hiking, jogging, biking, exercise cycling, dancing, aerobics, bowling, golf, singles tennis, doubles tennis, racquetball, calisthenics, swimming.  – Males: Kcals/week < 383  – Females: Kcals/week < 270 |
| **3. Presence of Frailty**  Positive for frailty phenotype: ≥ 3 criteria present  Intermediate or prefrail: 1 or 2 criteria present | |

Supplementary Table 2. Characteristics of physical frailty phenotype of fifteen older adults.

|  | **Gender** | **Age**  **(Years old)** | **Shrinking** | **Weakness** | **Exhaustion** | **Slowness** | **Low activity** |
| --- | --- | --- | --- | --- | --- | --- | --- |
| **non-frail older adults** | M | 87 | × | × | × | × | × |
|  | M | 81 | × | × | × | × | × |
|  | M | 83 | × | × | × | × | × |
|  | M | 79 | × | × | × | × | × |
|  | M | 82 | × | × | × | × | × |
| **pre-frail older adults** | F | 80 | × | √ | × | × | √ |
|  | M | 81 | √ | √ | × | × | × |
|  | F | 82 | × | × | √ | × | × |
|  | M | 90 | × | √ | × | × | × |
|  | M | 85 | × | √ | × | × | √ |
| **frail older adults** | F | 77 | √ | × | √ | × | √ |
|  | M | 90 | × | √ | √ | √ | √ |
|  | M | 84 | × | √ | × | √ | √ |
|  | F | 87 | × | √ | √ | √ | √ |
|  | M | 80 | × | √ | √ | √ | √ |

Supplementary Table 3. Characteristics of study participants according to frail status.

|  | **Middle-aged** | **Non-frail** | | **Pre-fail** | **Frail** | ***P* value** | | | |
| --- | --- | --- | --- | --- | --- | --- | --- | --- | --- |
|  | **(Group A)** | **(Group B)** | **(Group C)** | | **(Group D)** | **A vs B** | **B vs C** | **B vs D** | **C vs D** |
| **Age, years** | 44(40-48) | 82(80-85) | 82(80.5-87.5) | | 84(79-88.5) | 0.009** | 0.753 | 0.599 | 0.916 |
| **Sex, M/F** | 3/2 | 5/0 | 3/2 | | 3/2 | 0.444 | 0.444 | 0.444 | 1.000 |
| **BMI, kg/m^2^** | 21.80  (20.86-27.41) | 23.67  (22.67-25.77) | 22.89  (19.91-24.24) | | 24.35  (23.33-25.67) | 0.465 | 0.347 | 0.465 | 0.117 |
| **Health behavior** | | | | | | | | | |
| Smoking | 2(40%) | 1(20%) | 0(0%) | | 1(20%) | 1.000 | 1.000 | 1.000 | 1.000 |
| Alcohol drinking | 2(40%) | 1(20%) | 0(0%) | | 1(20%) | 1.000 | 1.000 | 1.000 | 1.000 |
| **Total number of comorbidities** [N**o. (%)]** | 1(0-1.5) | 1(0.5-2.5) | 4(2.5-5) | | 4(2-5.5) | 0.381 | 0.043* | 0.044* | 0.914 |
| **Medications** | | | | | | | | | |
| Total number of. medications [No. (%)] | 0(0-0.5) | 1(0.5-2) | 4(1.5-4.5) | | 4(3.5-7.5) | 0.065 | 0.109 | 0.011* | 0.28 |
| Psycho-drugs | 0(0%) | 0(0%) | 0(0%) | | 2(40%) | 1 | 1 | 0.444 | 0.444 |
| Antidiabetic agent | 0(0%) | 0(0%) | 1(20%) | | 4(80%) | 1 | 1 | 0.048 | 0.206 |
| Statin | 0(0%) | 2(40%) | 0(0%) | | 1(20%) | 0.444 | 0.444 | 1 | 1 |
| β-blocker | 0(0%) | 0(0%) | 1(20%) | | 0(0%) | 1 | 1 | 1 | 1 |
| Antiplatelet | 0(0%) | 1(20%) | 1(20%) | | 1(20%) | 1 | 1 | 1 | 1 |
| ACEi and/or ARB | 1(20%) | 1(20%) | 1(20%) | | 3(60%) | 1 | 1 | 0.524 | 0.524 |
| β2 sympathomimetic inhaler | 0(0%) | 0(0%) | 1(20%) | | 2(40%) | 1 | 1 | 0.444 | 1 |

Binary data was presented as number (percentage). Continuous variables were presented as median (interquartile range). * *p* value < 0.05, ** *p* value < 0.01.

Supplementary Table 13. Relative abundance of fecal microbiota at the genus level.

| **Bacteria species** | **Middle-aged group** | **Non-frail group** | ***P* value** |
| --- | --- | --- | --- |
|  | Relative abundance（%） | |  |
| Clostridium_XVIII | 0.389468 | 0.016432 | 0.036714 |
| Clostridium_XlVb | 1.013768 | 0.188664 | 0.036714 |
| **Bacteria species** | **Non-frail group** | **Frail group** | ***P* value** |
|  | Relative abundance（%） | |  |
| Faecalibacterium  (Faecalibacterium prausnitzii) | 8.672279 | 0.023594 | 0.011925 |
| Bacteroides | 9.051243 | 29.700802 | 0.036714 |
| Fusicatenibacter | 0.134094 | 0.009723 | 0.034454 |
| Parabacteroides | 0.211326 | 7.703806 | 0.012186 |
| Roseburia | 3.396313 | 0.055512 | 0.012186 |

Supplementary Table 14. Spearman’s rank correlations between the altered microbiota at genus level and fecal or serum metabolites in frailty (non-frail group vs. frail group).

|  | | **Bacteroides** | | **Faecalibacterium** | | **Fusicatenibacter** | | **Parabacteroides** | | **Roseburia** | |
| --- | --- | --- | --- | --- | --- | --- | --- | --- | --- | --- | --- |
|  |  | r | p | r | p | r | p | r | p | r | p |
| **Fecal metabolites** | **2'-deoxyinosine** | -0.48 | 0.16 | 0.48 | 0.16 | 0.48 | 0.16 | -0.70 | 0.03 | 0.40 | 0.25 |
|  | **Creatine** | 0.58 | 0.08 | -0.58 | 0.08 | -0.68 | 0.03 | 0.70 | 0.03 | -0.58 | 0.08 |
|  | **4-methylphenol** | -0.58 | 0.08 | 0.75 | 0.01 | 0.56 | 0.09 | -0.77 | 0.01 | 0.76 | 0.01 |
|  | **Tyramine** | -0.81 | 0.00 | 0.93 | 0.00 | 0.82 | 0.00 | -0.84 | 0.00 | 0.69 | 0.03 |
|  | **Indole-3-ethanol** | 0.78 | 0.01 | -0.80 | 0.01 | -0.69 | 0.03 | 0.65 | 0.04 | -0.43 | 0.22 |
|  | **Dodecanedioic acid** | -0.82 | 0.01 | 0.86 | 0.00 | 0.74 | 0.01 | -0.65 | 0.049 | 0.77 | 0.01 |
| **Serum metabolites** | **D-ribose** | 0.55 | 0.10 | -0.75 | 0.01 | -0.77 | 0.01 | 0.73 | 0.02 | -0.53 | 0.12 |
|  | **D-(-)-mannitol** | 0.72 | 0.02 | -0.82 | 0.00 | -0.79 | 0.01 | 0.94 | 0.00 | -0.63 | 0.05 |
|  | **Creatine** | -0.39 | 0.26 | 0.64 | 0.047 | 0.51 | 0.13 | -0.73 | 0.02 | 0.78 | 0.01 |
|  | **Indole** | -0.84 | 0.00 | 0.81 | 0.00 | 0.85 | 0.00 | -0.95 | 0.00 | 0.58 | 0.08 |
|  | **Dodecanedioic acid** | -0.87 | 0.00 | 0.77 | 0.01 | 0.78 | 0.01 | -0.58 | 0.09 | 0.59 | 0.08 |

Supplementary Table 15. Spearman’s rank correlations between the altered fecal metabolites and serum metabolites in frailty (non-frail group vs. frail group).

|  | | **Fecal metabolites** | | | | | | | | | |
| --- | --- | --- | --- | --- | --- | --- | --- | --- | --- | --- | --- |
|  |  | **2'-deoxyinosine** | | **Creatine** | | **4-methylphenol** | | **Tyramine** | | **Indole-3-ethanol** | |
|  |  | r | p | r | p | r | p | r | p | r | p |
| **Serum metabolites** | **D-ribose** | -0.76 | 0.02 | 0.59 | 0.08 | -0.72 | 0.02 | -0.72 | 0.02 | 0.75 | 0.02 |
|  | **D-(-)-mannitol** | -0.79 | 0.01 | 0.62 | 0.06 | -0.83 | 0.01 | -0.73 | 0.02 | 0.76 | 0.02 |
|  | **Creatine** | 0.32 | 0.37 | -0.68 | 0.04 | 0.65 | 0.049 | 0.71 | 0.03 | -0.21 | 0.56 |
|  | **Indole** | 0.48 | 0.17 | -0.79 | 0.01 | 0.72 | 0.02 | 0.92 | 0.00 | -0.72 | 0.02 |
